# Isolation and Characterization of Two Bacteriophages and Their Preventive Effects against Pathogenic *Vibrio coralliilyticus* Causing Mortality of Pacific Oyster (*Crassostrea gigas*) Larvae

**DOI:** 10.3390/microorganisms8060926

**Published:** 2020-06-19

**Authors:** Hyoun Joong Kim, Sib Sankar Giri, Sang Guen Kim, Sang Wha Kim, Jun Kwon, Sung Bin Lee, Se Chang Park

**Affiliations:** Laboratory of Aquatic Biomedicine, College of Veterinary Medicine and Research Institute for Veterinary Science, Seoul National University, Seoul 08826, Korea; hjoong1@nate.com (H.J.K.); giribiotek@gmail.com (S.S.G.); imagine5180@gmail.com (S.G.K.); kasey.kim90@gmail.com (S.W.K.); kjun1002@naver.com (J.K.); lsbin1129@naver.com (S.B.L.)

**Keywords:** Pacific oyster larvae, mass mortality, *Vibrio coralliilyticus*, bacteriophage, phage antibiotic substitution

## Abstract

*Vibrio coralliilyticus* is one of the major pathogens causing mass mortality in marine bivalve larvae aquaculture. To prevent and control *Vibrio* spp. infections in marine bivalve hatcheries, various antibiotics are overused, resulting in environmental pollution and the creation of multi-drug-resistant strains. Therefore, research on the development of antibiotic substitutes is required. In this study, we isolated two bacteriophages (phages) that specifically infected pathogenic *V. coralliilyticus* from an oyster hatchery and designated them as pVco-5 and pVco-7. Both phages were classified as *Podoviridae* and were stable over a wide range of temperatures (4–37 °C) and at pH 7.0–9.0. Thus, both phages were suitable for application under the environmental conditions of an oyster hatchery. The two phages showed confirmed significant bactericidal efficacy against pathogenic *V. coralliilyticus* in an in vitro test. In the in vivo experiment, the phage pre-treated groups of Pacific oyster larvae showed significantly lower mortality against *V. coralliilyticus* infection than untreated control larvae. The results of the present study suggest that both phages could be used in the artificial marine bivalve seedling industry; not only to prevent pathogenic *V. coralliilyticus* infection, but also to reduce antibiotic overuse.

## 1. Introduction

Asia has the highest yields of oysters in the world. In 2017 it produced 5,435,627 tons, 95.19% of the global production (5,710,522 tons) [1]. Since the development of marine bivalve artificial seed production technology, numerous countries have produced seedlings of various species. In Korea, the artificial seedling production industry for Pacific oysters has actively developed, mainly in the southern region. The Republic of Korea produced 315,255 tons of Pacific oysters (*Crassostrea gigas*), 49.33% of the global production (639,030 tons) [1], making it the largest producer of Pacific oysters in the world. However, since the mid-2000s, outbreaks of *Vibrio coralliilyticus* and Ostreid Herpesvirus-1 uVar (OsHV-1 uVar) have led to frequent mass mortalities of Pacific oysters in Korea [2,3,4,5].

*Vibrio* spp. has been reported to be one of the causative agents of the mass mortality of marine bivalve larvae. *V. coralliilyticus* and *V. tubiashii* are known to be major bacterial pathogens of marine bivalve larvae worldwide [5,6,7,8,9,10]. To prevent and control *Vibrio* spp. infection, application of various antibiotics has been suggested [8,11,12]. *V. coralliilyticus* is one of major causative agents of the mass mortality of marine bivalve larvae [5,7,8]. It has been reported to show multi-drug-resistance to various antibiotics, including vancomycin, oxacillin, cefaperazolin, ampicillin, ampicillin-sulbactam, piperacillin-tazobactam, cefepime, cefotaxime, cefoxitin, ceftazidime, and meropenem [4,8]. Matsubara et al. [12] reported that chloramphenicol, gentamicin, streptomycin, and erythromycin showed therapeutic efficacy against *V. coralliilyticus* strain 60, previously identified as *V. splendidus* biovar II but re-identified as *V. coralliilyticus* by Kim et al. [13], in in vivo tests using Pacific oyster larvae. However, while antibiotics show significant bactericidal effects, their overuse can also lead to a generation of multiple-antibiotic-resistant bacteria, environmental pollution, and the destruction of beneficial bacterial populations [14,15]. Therefore, various studies have attempted to identify substitutes for antibiotics such as bacteriophages, probiotics, and egg extracts in marine invertebrate aquaculture [16,17,18,19,20,21,22,23]. 

A bacteriophage (phage) is a virus that can infect only bacteria. Unlike antibiotics that kill all bacteria, including beneficial species, lytic phages infect and kill only targeted bacteria. Therefore, phages are being actively studied as a viable substitute for antibiotics [24,25,26]. Phages have also been used in aquaculture to control bacterial diseases [16,17,18,19,27,28,29,30]. 

The present study is aimed at preventing mass mortalities in oyster hatcheries and identifying antibiotic substitutes by isolating and characterizing two lytic phages infecting *V. coralliilyticus*. We also assessed the preventive effects of each phage against *V. coralliilyticus* infection in in vitro tests and an in vivo test using Pacific oyster larvae. 

## 2. Materials and Methods

### 2.1. Bacterial Strains and Growth Conditions

In this study, 12 bacterial strains were used, including four *V. coralliilyiticus* strains that were previously isolated from moribund Pacific oyster larvae in Japan [8], and seven strains of other *Vibrio* species. A reference strain, *Escherichia coli* (ATCC 25922), was used as the control (Table 1). *V. coralliilyticus* 58 (designated Vco58), which has been reported to show high virulence for Pacific oyster larvae [8,19], was used as the host strain to isolate the phages. Sodium chloride (final concentration, 2.0%), added tryptic soy agar (TSA; BD Difco, Sparks, MD, USA), tryptic soy broth (TSB; BD Difco, Sparks, MD, USA), and TSB top agar were used for bacterial culture and phage preparation.

### 2.2. Phage Isolation, Purification, and Propagation

Seawater samples were collected from oyster hatchery environments such as larvae tanks, broodstock tanks, algae tanks, sewage, and the coastal areas of Goseong and Goheung, which are located in the southern region of Korea. Each seawater sample (450 mL) was filtered using 0.45-µm pore-size membrane filters. The samples were then gently mixed with 50 mL of 10X TSB and a colony of Vco58 cultured on TSA. The mixture was incubated in a shaking incubator at 27 °C at 150 rpm for 24 h. The enriched culture was centrifuged at 13,000 × *g* for 10 min, and the supernatant was filtered through a 0.22-µm pore-size membrane filter. To confirm phage isolation, a spot assay was performed using the protocol previously described by Cerveny et al. [36]. To obtain pure phage, the isolated phages were cloned via a double-layered agar method with a single plaque in triplicate [37]. The CsCl density gradient method was performed to obtain the purified and concentrated phage [38]. The phage titer was determined by calculating the plaque-forming units per milliliter (PFU/mL) of phage using the double-layered agar method.

### 2.3. Phage Characterization

#### 2.3.1. Host Range and Efficiency of Plating (EOP)

Vco58, the strain showing high virulence for Pacific oyster larvae, was used as the standard strain for phage isolation and as an EOP value indicator. The infectivity of the isolated phages was tested using 12 bacterial strains via spot assays (Table 1). In these assays, 10 µL of cloned and purified phage solution (pVco-5: 8.2 × 10^8^ PFU/mL, pVco-7: 1.52 × 10^8^ PFU/mL) was dropped onto each bacterial lawn on a TSA plate and incubated at the optimal temperature of each strain for 24 h. After incubation, the plates were checked for the presence of plaques, and the EOP value was calculated by determining the PFU ratio of each susceptible strain against the standard strain through the double-layered agar method and conducted in triplicate.

#### 2.3.2. Electron Microscopy

Purified phage lysates (≥ 10^9^ PFU/mL) obtained via the CsCl density gradient method [38] were negatively stained using 2% uranyl acetate onto a copper grid for 1 min, which was followed by three successive washes with double-distilled water. After washing, the grid was dried in a desiccator for 10 min, and the dried grid was observed with a transmission electron microscope (TEM), Talos L120C (FEI, Hillsboro, Ore, USA) operating at 120 kV. The sizes of the phages observed through the TEM were measured using an image analysis program (Motic Image Plus 2.0, Xiamen, China). 

#### 2.3.3. Phage Stability Test 

Phage stability under various pH and thermal conditions was assessed as previously described by Verma et al. [39]. For stability tests under different pH conditions, the pH of 10^8^ PFU/mL (pVco-5: 8.2 × 10^8^ PFU/mL, pVco-7: 1.52 × 10^8^ PFU/mL) phage suspensions was set to 3, 5, 7, 9, and 11 using 1 M NaOH and 1 M HCl. After pH adjustment, each phage suspension was incubated at 27 °C for 1 h, and the phage titer was subsequently calculated via the double-layered agar method. To evaluate stability under different thermal conditions, 10^8^ PFU/mL phage suspensions were incubated at 4, 15, 20, 27, 37, 50, and 60 °C for 1 h, and the phage titer was subsequently calculated.

#### 2.3.4. Host Cell Lysis Test

To evaluate the bactericidal effect of each isolated phage, Vco58 was used as the host. The bacterial cell lysis test was performed as previously described [19]. The multiplicity of infections (MOI) of each phage was adjusted to 0, 0.1, 1, and 10 with pure phage administration in the early exponential phase of Vco58 (OD_600_ value: 0.042). Absorbance (OD_600_) was monitored at 3 h intervals for 24 h. All tests were performed with six replicates.

### 2.4. Prophylactic Efficacy of the Isolated Phages

To eliminate the nutrient content in phage lysates for the in vivo test, phage suspensions were refined using 10% (wt /vol) polyethylene glycol 8000 and 1 M sodium chloride in accordance with the protocol proposed by Kim et al [17]. The experimental conditions used for determining the prophylactic efficacy of phages are shown in Table 2. Oyster larvae (*n* = 5 ± 1.6 /mL) were incubated into 6-well plates with 8 mL of filtered and sterilized seawater (FSS). One milliliter of each refined phage lysate, adjusted to 10^5^, 10^6^, 10^7^, and 10^8^ PFU/mL (pVco-5: 1.25 × 10^*n*^ and pVco-7: 1.44 × 10^*n*^), was inoculated into each well and acclimated for 1 h at 27 °C (phage concentration in each well was adjusted to 10^4^, 10^5^, 10^6^, or 10^7^ PFU/mL). Subsequently, 1 mL of Vco58 culture (1.87 × 10^6^ CFU/mL) washed three times with FSS was inoculated into each well and incubated at 27 °C for 24 h. We observed the cumulative mortality of oyster larvae in each well at 6 h intervals. Larvae without the cilia and intravalvular movement were determined dead following the protocol previously described by Sugumar et al. [8]. Control wells containing bacteria without phage inoculation or phages without bacterial inoculation were created. Each sample was assessed in triplicate under the same conditions.

### 2.5. Statistical Analysis

Statistical analysis was conducted using SigmaPlot version 14.0 software (Systat Software, Inc. Chicago, IL, USA). The one-way analysis of variance (ANOVA) was used to analyze the data followed by the Bonferroni post-hoc test. A *p* value of <0.05 was considered statistically significant.

## 3. Results

### 3.1. Phage Isolation and Morphology

**Figure 1:**
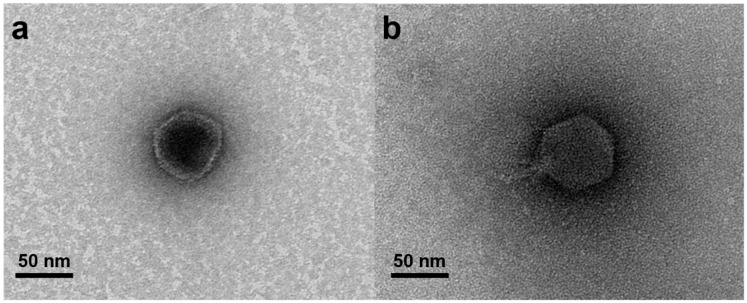
Electron micrograph of isolated phages, pVco-5 (**a**) and pVco-7 (**b**).

Two phages, designated pVco-5 and pVco-7, specifically infecting Vco58 were isolated from the broodstock tank water of the oyster hatchery located at Goheung (pVco-5) and the larvae tank water of the oyster hatchery located at Goseong (pVco-7) in Korea. Both phages showed bactericidal effects against the four kinds of *V. coralliilyticus* strains used in this study (Table 1). In TEM assessments, pVco-5 and pVco-7 showed an icosahedral capsid. The average diameter of pVco-5 was 54.96 ± 2.07 nm (*n* = 20) and that of pVco-7 was 60.71 ± 3.55 nm. Both pVco-5 and pVco-7 showed an icosahedral capsid with a short non-contractile tail. This means that both phages are classified as *Podoviridae* based on the morphological classification system proposed by Ackermann [40] (Figure 1).

### 3.2. Characterization of pVco-5 and pVco-7

#### 3.2.1. Host Range and EOPs

In the host range test, eleven *Vibrio* spp., including four *V. coralliilyticus* strains, and *E. coli* were tested to evaluate the inhibition activity of pVco-5 and pVco-7. Both phages inhibited Vco58 and other *V. coralliilyticus* strains, whereas the other *Vibrio* spp. and *E. coli* were not susceptible to pVco-5 and pVco-7 (Table 1). In assessments of the EOP values of pVco-5 and pVco-7, Vco58 was used as the standard strain, and the other three strains of *V. coralliilyticus* showed very similar EOP values to that of Vco58. These findings confirmed that both phages showed very similar inhibition effects on the *V. coralliilyticus* strains tested in this study.

#### 3.2.2. Stability Test

**Figure 2:**
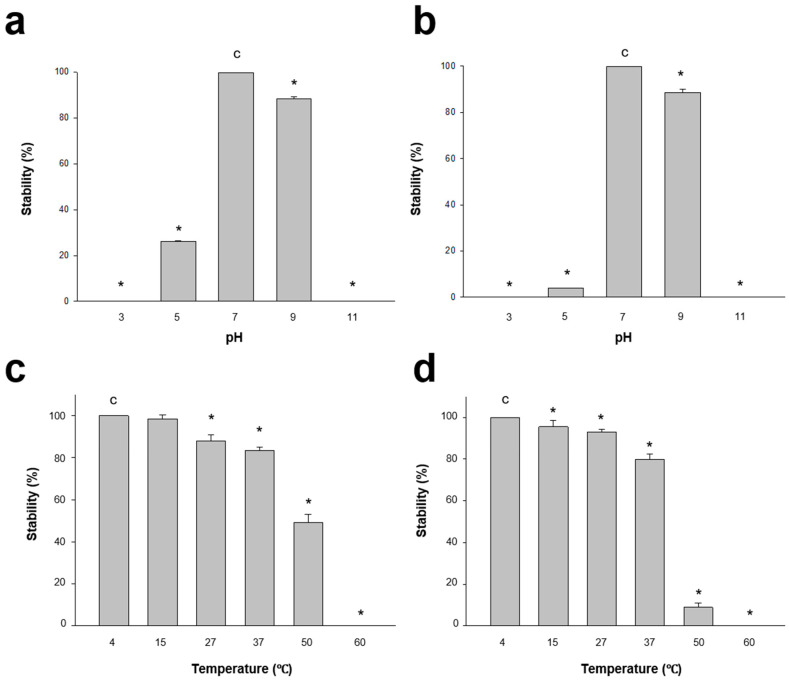
Stability of pVco-5 (**a**,**c**) and pVco-7 (**b**,**d**) in the presence of various pHs and temperatures. 4 °C and pH 7 were used as controls. Asterisks means *p* < 0.001 against control (**c**).

The stability of both phages under various pH conditions was assessed, and pVco-5 was shown to be very stable at pH 7 (100%) and pH 9 (88.5% ± 0.85%). However, it exhibited 26.1% ± 0.41% stability at pH 5 and 0% activity at pH 3 and pH 11 (Figure 2a). Similarly, pVco-7 also did not exhibit activity at pH 3 and pH 11 and showed only 3.97% ± 0.05% stability at pH 5. At pH values of 7 and 9, it showed 100% and 88.7% ± 1.27% stability, respectively (Figure 2b). Both phages showed similar stability patterns at various pH values, but in an acid environment (pH 5), pVco-5 was 20% more stable than pVco-7. In assessments performed under different thermal conditions, pVco-5 was stable at 4–37 °C, and its activity reduced with increasing temperature (100% at 4 °C, 98.46% ± 1.75% at 15 °C, 87.93% ± 2.79% at 27 °C, 83.41% ± 1.74% at 37 °C, 48.97% ± 4.15% at 50 °C, and 0% at 60 °C) (Figure 2c). pVco-7 also showed similar thermal stability patterns to pVco-5, with over 90% at 4–27 °C and reduced activity at higher temperatures (100% at 4 °C, 95.44% ± 3.16% at 15 °C, 92.84% ± 1.54% at 27 °C, 79.86% ± 2.43% at 37 °C, 8.89% ± 2.25% at 50 °C, and 0% at 60 °C) (Figure 2d). pVco-5 showed over 40% stability at 50 °C in comparison with pVco-7.

#### 3.2.3. Bacterial Cell Lysis Test

**Figure 3:**
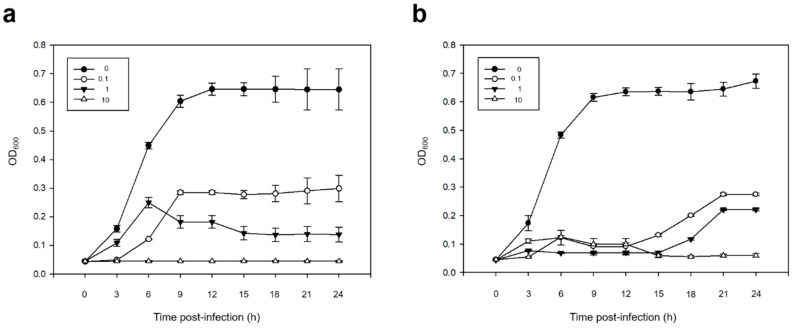
Cell lysis effect of pVco-5 (**a**) and pVco-7 (**b**) against *V. coralliilyticus* 58 in various multiplicities of infections (MOIs). As a result of statistical analysis, the *p* value for each hour was verified to be less than 0.001 when both pVco-5 and pVco-7 were compared to the control group (MOI: 0).

The results for the bactericidal effects of pVco-5 and pVco-7 against Vco58 are shown in Figure 3. The OD_600_ values of all the control groups (MOI: 0) showed an identical continuous increase during the incubation period. In the case of pVco-5, no increase in the OD_600_ value was observed in the Vco58-treated group when the MOI was 10, but when MOI was 1, the OD_600_ value slightly increased until 6 h and decreased gradually thereafter (Figure 3a). When the lowest MOI (0.1) was applied, Vco58 growth reached OD_600_ = 0.285 ± 0.006 after 6 h of exposure and showed partial lysis. In the case of pVco-7, Vco58 growth was observed until 12 h (OD_600_ = 0.099 ± 0.004), but it gradually decreased to 0.059 ± 0.006 with cell lysis after 24 h when the MOI was 10 (Figure 3b). When MOI was 1 and 0.1, low OD_600_ values were noted in comparison to the control group due to the cell lysis activity of the phage. Both phages showed significant increments in bactericidal efficacy with increasing phage concentrations.

### 3.3. Preventive Efficacy of Pre-phage Treatment

**Figure 4:**
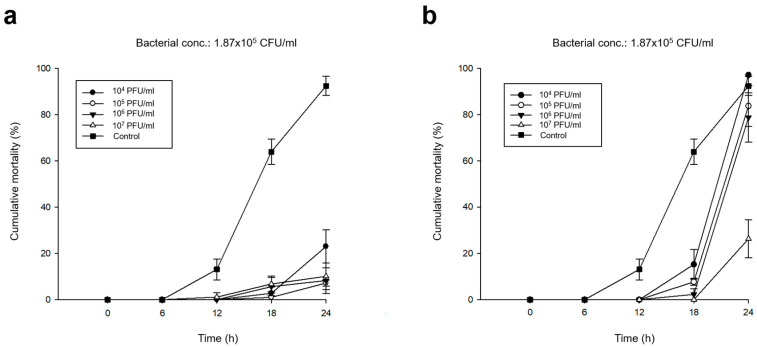
Prophylactic efficacy of various concentrations of pVco-5 (**a**, 1.25 × 10^n^ PFU/mL) and pVco-7 (**b**, 1.44 × 10^n^ PFU/mL) against Vco58 (conc.: 1.87 × 10^5^ CFU/mL) in Pacific oyster larvae. As a result of statistical analysis, all groups except the 10^4^ PFU/ml of pVco-7 treated group at 24 h were shown *p* < 0.001 when compared with the control group.

The results outlining the prophylactic effect of each phage treatment are shown in Table 2 and Figure 4. The phage-untreated control showed 92.49%±4.12% cumulative mortality within 24 h. In contrast, the pVco-5-treated groups showed significantly higher survival rates than the untreated control. When phage concentrations were 1.25 × 10^5^ to 1.25 × 10^7^ PFU/mL, cumulative mortality rates were below 11%, and when the phage concentration was 1.25 × 10^4^ PFU/mL, the mortality rate was 23.00% ± 7.20% (Figure 4a). Meanwhile, pVco-7 treatment at a phage concentration of 1.44 × 10^7^ PFU/mL resulted in significant survival rates (26.32% ± 8.14%). However, the other phage-treated groups (1.44 × 10^4^ to 1.44 × 10^6^ PFU/mL) did not show much difference from the untreated control. Thus, pVco-5 showed greater preventive efficacy than pVco-7 in the in vivo tests.

## 4. Discussion

Phages are viruses that invade bacteria, and lytic phages are widely used as biocontrol agents because of their bactericidal activity. A phage has a narrow host range and only infects its target bacterial species. Thus, it could be good candidate for the inhibition of specific harmful bacteria. Therefore, phage application is being actively investigated in various fields, including aquaculture, to replace the overuse of antibiotics and eliminate multiple-antibiotic-resistant bacteria [17,18,20,27,28,29,30]. For these reasons, the present study also focused on the use of phages to prevent the mass mortality caused by the *V. coralliilyticus* infection in marine bivalve hatcheries. 

Both isolated phages pVco-5 and pVco-7 infected only four virulent *V. coralliilyticus* strains and did not infect other *Vibrio* spp. and the *E. coli* strain tested in this study. pVco-5 showed similar infectivity against four *V. coralliilyticus* strains, but the EOP value of pVco-7 against the *V. coralliilyticus* Q1 strain was almost half of that recorded against other strains (Table 1). 

To confirm the preventive efficacy of pVco-5 and pVco-7, in vivo tests were conducted using Pacific oyster larvae. In these tests, pVco-5-treated larvae showed significantly higher survival rates than untreated control larvae (Figure 4a). Thus, pVco-5 could be an excellent preventive agent against *V. coralliilyiticus* infection in marine bivalve hatcheries. In contrast, pVco-7 showed a strong preventive effect only at phage concentrations of 1.44 × 10^7^ PFU/mL (MOI: 100), while the larvae treated with pVco-7 concentrations of 1.44 × 10^4^ to 1.44 × 10^6^ PFU/mL (MOI: 0.1, 1 and 10) showed a similar cumulative mortality as the untreated controls (Figure 4b). However, phages are very easy to mass produce at high concentrations. Therefore, pVco-7 could also be used as an effective preventive agent against *V. coralliilyticus* because it showed a very high preventive efficacy at high titers. In the case of the cell lysis test result, both phages showed considerable bactericidal effects at MOI 10 and, when MOIs were 1 and 0.1, a weaker inhibitory effect was observed. When comparing the results of the cell lysis test and the prophylactic efficacy test, the effect of each MOI was not consistent. This suggests that, unlike the bactericidal effect in the direct relationship between bacteria and phages, the bactericidal effect of phages may be different when mediating oyster larvae is included.

Both phages were very stable at pH 7–9 and over a temperature range of 4 °C to 37 °C. Pacific oyster larvae are usually cultured at 24–28 °C and pH 7–9 in seedling hatcheries. Thus, pVco-5 and pVco-7 are suitable for larval culture environments and can be used as a preventive agent against *V. coralliilyticus* infection in seedling hatcheries.

Since the development of artificial seedling cultures, the production of marine bivalves has been increasing worldwide. However, pathogens such as *V. coralliilyticus*, *V. tubiashii*, and OsHV-1 can cause substantial economic losses [4,5,8,9,10,41]. Unlike vertebrates, bivalves do not have an acquired immune system, ruling out the use of agents such as vaccines and severely limiting the options for prevention and treatment of infections. In this study, phage application was proposed to overcome the problems associated with antibiotics, such as the occurrence of multiple-antibiotic-resistant bacteria, destruction of beneficial microbial populations, and contamination of the environment. Both isolated phages were suitable for application in the seedling hatchery environment, and the in vivo test results showed strong prophylactic efficacy in the prevention of the *V. coralliilyticus* strains tested in this study. 

In conclusion, the phages isolated in this study showed extremely effective activity for the prevention of pathogenic *V. coralliilyticus*, which causes mass mortality in marine bivalve seedling production, suggesting that they could be used as antibiotic substitutes in the future.

## Figures and Tables

**Table 1:** Bacterial Strains Used in This Study and Infectivity of Isolated Bacteriophage (Phage) pVco-5 and pVco-7.

Bacterial Species	Strain	Host Range ^a^ (EOPs ^b^)		Source ^a^
		pVco-5	pVco-7	
*V. coralliilyticus*	58	+(1)	+(1)	[8]
*V. coralliilyticus*	59	+(0.94 ± 0.75)	+(1 ± 0.12)	[8]
*V. coralliilyticus*	60	+(1.03 ± 0.13)	+(0.91 ± 0.09)	[8]
*V. coralliilyticus*	Q1	+(0.9 ± 0.44)	+(0.53 ± 0.12)	[8]
*V. tubiashii*	ATCC19109	-	-	ATCC
*V. parahaemolyticus*	CRS09-17	-	-	[31]
*V. alginolyticius*	rM8402	-	-	[32]
*V. anguillarum*	HT7602	-	-	[33]
*V. cholerae*	PS-7701	-	-	[34]
*V. harveyi*	ATCC14126	-	-	ATCC
*V. vulnificus*	ET7618		-	[35]
*Echerichia coli*	ATCC25922	-	-	ATCC

^a^ +, susceptible; not susceptible; ^b^ mean ± SD.

**Table 2:** Experimental Conditions and In Vivo Test Result of Prophylactic Efficacy of Pre-phage Treatment Against Vco 58 Infection.

Phage	Phage conc. (PFU/mL)	Vco 58 conc. (CFU/mL)	Mortality (24 h Post-Infection) (%, Mean ± SD)
pVco-5	1.25 × 10^4^	1.87 × 10^5^	23.00 ± 7.20
	1.25 × 10^5^	1.87 × 10^5^	7.18 ± 1.28
	1.25 × 10^6^	1.87 × 10^5^	8.25 ± 5.55
	1.25 × 10^7^	1.87 × 10^5^	10.09 ± 5.71
pVco-7	1.44 × 10^4^	1.87 × 10^5^	97.20 ± 1.21
	1.44 × 10^5^	1.87 × 10^5^	83.80 ± 8.90
	1.44 × 10^6^	1.87 × 10^5^	78.85 ± 10.67
	1.44 × 10^7^	1.87 × 10^5^	26.32 ± 8.14
Control	0	1.87 × 10^5^	92.49 ± 4.12

## References

[1] FAO Fish Stat (2019). Global Aquaculture Production for Species (Tonnes): Pacific Oyster. http://www.fao.org/fishery/topic/16140/en.

[2] Jee B.Y., Lee S.J., Cho M.Y., Lee S.J., Kim J.W., Choi S.H., Kim K.H. (2013). Detection of Ostreid Herpesvirus 1 from adult Pacific oysters *Crassostrea gigas* cultured in Korea. Fish. Aquat. Sci..

[3] Hwang J., Park J., Yu H., Hur Y., Arzul I., Couraleau Y., Park M. (2013). Ostreid herpesvirus 1 infection in farmed Pacific oyster larvae *Crassostrea gigas* (Thunberg) in Korea. J. Fish Dis..

[4] Kim H.J., Jun J.W., Giri S.S., Yun S., Kim S.G., Kim S.W., Kang J.W., Han S.J., Kwon J., Oh W.T. (2019). Mass mortality in Korean bay scallop (*Argopecten irradians*) associated with Ostreid Herpesvirus-1 uVar. Transbound. Emerg. Dis..

[5] Kim H.J., Jun J.W., Giri S.S., Chi C., Yun S., Kim S.G., Kim S.W., Han S.J., Kwon J., Oh W.T. (2020). Identification and genome analysis of *Vibrio coralliilyticus* causing mortality of Pacific oyster (*Crassostrea gigas*) larvae. Pathogens.

[6] Elston R.A., Hasegawa H., Humphrey K.L., Polyak I.K., Häse C.C. (2008). Re-emergence of Vibrio tubiashii in bivalve shellfish aquaculture: Severity, environmental drivers, geographic extent and management. Dis. Aquat. Org..

[7] Richards G.P., Watson M.A., Needleman D.S., Church K.M., Häse C.C. (2015). Mortalities of Eastern and Pacific oyster larvae caused by the pathogens *Vibrio coralliilyticus* and *Vibrio tubiashii*. Appl. Environ. Microbiol..

[8] Sugumar G., Nakai T., Hirata Y., Matsubara D., Muroga K. (1998). *Vibrio splendidus biovar* II as the causative agent of bacillary necrosis of Japanese oyster *Crassostrea gigas* larvae. Dis. Aquat. Org..

[9] Travers M.-A., Achour R.M., Haffner P., Tourbiez D., Cassone A.-L., Morga B., Fruitier-Arnaudin I. (2014). First description of French *V. tubiashii* strains pathogenic to mollusk: I. Characterization of isolates and detection during mortality events. J. Invertebr. Pathol..

[10] Tubiash H.S., Chanley P.E., Leifson E. (1965). Bacillary necrosis, a disease of larval and juvenile bivalve mollusks I. Etiology and epizootiology. J. Bacteriol..

[11] Dubert J., Osorio C.R., Prado S., Barja J.L. (2016). Persistence of antibiotic resistant *Vibrio* spp. in shellfish hatchery environment. Microb. Ecol..

[12] Matsubara D., Tanaka M., Soumyou Y., Hirakawa K., Doi R., Nakai T. (2002). Therapeutic effects of antimicrobial compounds against bacillary necrosis of larval Pacific oyster. Fish. Pathol..

[13] Kim H.J., Kim J.H., Jun J.W., Giri S.S., Chi C., Yun S., Kim S.G., Kim S.H., Kang J.W., Jeong D.G. (2017). Complete genome sequence of *Vibrio coralliilyticus* 58, Isolated from Pacific oyster (*Crassostrea gigas*) larvae. Genome Announc..

[14] Defoirdt T., Sorgeloos P., Bossier P. (2011). Alternatives to antibiotics for the control of bacterial disease in aquaculture. Curr. Opin. Microbiol..

[15] Giraud E., Blanc G., Bouju-Albert A., Weill F.X., Donnay-Moreno C. (2004). Mechanisms of quinolone resistance and clonal relationship among *Aeromonas salmonicida* strains isolated from reared fish with furunculosis. J. Med. Microbiol..

[16] Cohen Y., Joseph Pollock F., Rosenberg E., Bourne D.G. (2013). Phage therapy treatment of the coral pathogen *Vibrio coralliilyticus*. Microbiologyopen.

[17] Kim H.J., Jun J.W., Giri S.S., Chi C., Yun S., Kim S.G., Kim S.W., Kang J.W., Han S.J., Kwon J. (2019). Application of the bacteriophage pVco-14 to prevent *Vibrio coralliilyticus* infection in Pacific oyster (*Crassostrea gigas*) larvae. J. Invertebr. Pathol..

[18] Li Z., Li X., Zhang J., Wang X., Wang L., Cao Z., Xu Y. (2016). Use of phages to control *Vibrio splendidus* infection in the juvenile sea cucumber *Apostichopus japonicus*. Fish. Shellfish Immunol..

[19] Patil J.R., Desai S.N., Roy P., Durgaiah M., Saravanan R.S., Vipra A. (2014). Simulated hatchery system to assess bacteriophage efficacy against *Vibrio harveyi*. Dis. Aquat. Org..

[20] Jacquemot L., Bettarel Y., Monjol J., Corre E., Halary S., Desnues C., Bouvier T., Ferrier-Pages C., Baudoux A.-C. (2018). Therapeutic potential of a new jumbo phage that infects *Vibrio coralliilyticus*, a widespread coral pathogen. Front. Microbiol..

[21] Jun J.W., Han J.E., Giri S.S., Tang K.F., Zhou X., Aranguren L.F., Kim H.J., Yun S., Chi C., Kim S.G. (2018). Phage application for the protection from Acute Hepatopancreatic Necrosis Disease (AHPND) in *Penaeus vannamei*. Indian J. Microbiol..

[22] Kesarcodi-Watson A., Miner P., Nicolas J.-L., Robert R. (2012). Protective effect of four potential probiotics against pathogen-challenge of the larvae of three bivalves: Pacific oyster (*Crassostrea gigas*), flat oyster (*Ostrea edulis*) and scallop (*Pecten maximus*). Aquaculture.

[23] Takahashi K.G., Nakamura A., Mori K. (2000). Inhibitory Effects of Ovoglobulins on Bacillary Necrosis in Larvae of the Pacific Oyster, *Crassostrea gigas*. J. Invertebr. Pathol..

[24] Chhibber S., Kaur S., Kumari S. (2008). Therapeutic potential of bacteriophage in treating *Klebsiella pneumoniae* B5055-mediated lobar pneumonia in mice. J. Med. Microbiol..

[25] Kumari S., Harjai K., Chhibber S. (2009). Efficacy of bacteriophage treatment in murine burn wound infection induced by *Klebsiella pneumoniae*. J. Microbiol. Biotechnol..

[26] Kutter E., Sulakvelidze A. (2004). Bacteriophages: Biology and Applications.

[27] Hoai T.D., Mitomi K., Nishiki I., Yoshida T. (2018). A lytic bacteriophage of the newly emerging rainbow trout pathogen *Weissella ceti*. Virus Res..

[28] Jun J.W., Kim J.H., Shin S.P., Han J.E., Chai J.Y., Park S.C. (2013). Protective effects of the *Aeromonas* phages pAh1-C and pAh6-C against mass mortality of the cyprinid loach (*Misgurnus anguillicaudatus*) caused by *Aeromonas hydrophila*. Aquaculture.

[29] Park S.C., Nakai T. (2003). Bacteriophage control of *Pseudomonas plecoglossicida* infection in ayu *Plecoglossus altivelis*. Dis. Aquat. Org..

[30] Park S.C., Shimamura I., Fukunaga M., Mori K.I., Nakai T. (2000). Isolation of bacteriophages specific to a fish pathogen, *Pseudomonas plecoglossicida*, as a candidate for disease control. Appl. Environ. Microbiol..

[31] Jun J.W., Shin T.H., Kim J.H., Shin S.P., Han J.E., Heo G.J., De Zoysa M., Shin G.W., Chai J.Y., Park S.C. (2014). Bacteriophage therapy of a *Vibrio parahaemolyticus* infection caused by a multiple-antibiotic-resistant O3:K6 pandemic clinical strain. J. Infect. Dis..

[32] Takaoka O., Ji S.-C., Ishimaru K., Lee S.-W., Jeong G.-S., Ito J., Biswas A., Takii K. (2011). Effect of rotifer enrichment with herbal extracts on growth and resistance of red sea bream, *Pagrus major* (Temminck Schlegel) larvae against *Vibrio anguillarum*. Aquac. Res..

[33] Nakajima K., Muroga K., Hancock R.E.W. (1983). Comparison of fatty acid, protein, and serological properties distinguishing outer membranes of *Pseudomonas anguilliseptica* strains from those of fish pathogens and other Pseudomnonads. Int. J. Syst. Bacteriol..

[34] Austin B., Austin D.A., Blanch A.R., Cerda M., Grimont F., Grimont P.A.D., Jofre J., Koblavy S., Larsen J.L., Pedersen K. (1997). A comparison of methods for the typing of fish-pathogenic *Vibrio* spp.. Syst. Appl. Microbiol..

[35] Song Y.L., Lee S.P., Lin Y.T., Chen C.C. (1992). Enzyme immunoassay for shrimp vibriosis. Dis. Aquat. Org..

[36] Cerveny K.E., DePaola A., Duckworth D.H., Gulig P.A. (2002). Phage therapy of local and systemic disease caused by *Vibrio vulnificus* in iron-dextran-treated mice. Infect. Immun..

[37] Adams M.H. (1959). Bacteriophages.

[38] Sambrook J., Russell D.W., Russell D.W. (2001). Molecular cloning: A laboratory manual (3-volume set). Immunology.

[39] Verma V., Harjai K., Chhibber S. (2009). Characterization of a T7-like lytic bacteriophage of *Klebsiella pneumoniae* B5055: A potential therapeutic agent. Curr. Microbiol..

[40] Ackermann H.W. (2007). 5500 Phages examined in the electron microscope. Arch. Virol..

[41] Arzul I., Renault T., Lipart C. (2001). Experimental herpes-like viral infections in marine bivalves: Demonstration of interspecies transmission. Dis. Aquat. Org..

